# Entrapment of DNA topoisomerase-DNA complexes by nucleotide/nucleoside analogs

**DOI:** 10.20517/cdr.2019.95

**Published:** 2019-12-19

**Authors:** William H. Gmeiner

**Affiliations:** Department of Cancer Biology, Wake Forest School of Medicine, Winston-Salem, NC 27157, USA.

**Keywords:** DNA topoisomerase 1, cancer chemotherapy, cytarabine, gemcitabine, fluoropyrimidine

## Abstract

Topoisomerases are well-validated targets for cancer chemotherapy and DNA topoisomerase 1 (Top1) is the sole target of the camptothecin (CPT) class of anticancer drugs. Over the last 20 years, multiple studies have shown Top1 activity is modulated by non-native DNA structures and this can lead to trapping of Top1 cleavage complexes (Top1cc) and conversion to DNA double strand breaks. Among the perturbations to DNA structure that generate Top1cc are nucleoside analogs that are incorporated into genomic DNA during replication including cytarabine, gemcitabine, and 5-fluoro-2′-deoxyuridine (FdU). We review the literature summarizing the role of Top1cc in mediating the DNA damaging and cytotoxic activities of nucleoside analogs. We also summarize studies demonstrating distinct differences between Top1cc induced by nucleoside analogs and CPTs, particularly with regard to DNA repair. Collectively, these studies demonstrate that, while Top1 is a common target for both Top1 poisons such as CPT and nucleoside analogs such as FdU, these agents are not redundant. In recent years, studies have shown that Top1 poisons and nucleoside analogs together with other anti-cancer drugs such as cisplatin cause replication stress and the DNA repair pathways that modulate the cytotoxic activities of these compounds are being elucidated. We present an overview of this evolving literature, which has implications for how targeting of Top1 with nucleoside analogs can be used more effectively for cancer treatment.

## INTRODUCTION

Targeting DNA is one of the all-time most successful strategies for cancer treatment. Radiation and DNA damaging drugs such as cyclophosphamide have remained among the most widely used therapeutic modalities for cancer treatment for decades. A major advance for targeting DNA for cancer treatment was the discovery that camptothecin (CPT), a natural product identified by Wall and Wani^[1]^ in a screen for natural products with anti-cancer activity, targeted DNA topoisomerase 1 (Top1)^[2]^. In human cells, Top1, a TypeIB topoisomerase^[3]^, relieves superhelical density generated during replication^[4]^ and transcription^[5]^ by nicking supercoiled DNA to allow for spontaneous relaxation of supercoils (i.e., “controlled rotation”^[6]^), and then re-ligating the relaxed DNA. CPT analogs stabilize the nicked DNA with Top1 covalently bound in a ternary cleavage complex (Top1cc) that consists of Top1, DNA, and CPT^[7]^. The resulting Top1cc is converted into a DNA double strand break (DSB) upon collision with either an advancing replication fork^[8,9]^ or the transcriptional machinery^[10,11]^. Accumulation of unrepaired DNA DSBs stimulates activation of apoptosis.

With the realization that CPT analogs such as topotecan and Irinotecan (CPTs) displayed anti-cancer activity during clinical trials in the early 1990s^[12]^, there was growing appreciation that, in addition to its role as the sole target for CPTs, Top1 also mediated the cytotoxic effects of diverse treatments. These included generic DNA damage due to reactive oxygen species^[13,14]^, and nucleoside analogs^[15]^ used for cancer treatment. Studies from the Pommier lab showed that perturbations to DNA structure could result in either increased susceptibility to Top1-mediated DNA damage, or cause Top1 to be refractory from accessing sites with certain types of DNA damage. For example, abasic sites that may occur spontaneously in genomic DNA or as intermediates during base excision repair were shown to cause position-specific changes in Top1 cleavage activity^[16]^. Abasic sites within the first four bases 5’ to the Top1 cleavage site suppressed Top1 cleavage at the preferred site, but stimulated Top1 cleavage at alternative, nearby sites. However, an abasic site immediately 3’ to the Top1 cleavage site resulted in formation of a stable Top1cc even in the absence of CPT. Further studies showed that DNA with nicks or gaps with 5’-phosphate termini that could be generated by ionizing radiation or by the processing of abasic sites also modulated Top1 activity^[17]^. Nicks upstream of a preferred Top1 site suppressed Top1 activity, but irreversible Top1cc formed if nicks were positioned opposite to the Top1 cleavage site in the non-scissile strand. Top1 trapping was also detected at base mismatches^[16,18]^. These findings implicated Top1 in modulating repair of damaged DNA and indicated Top1cc could be intermediates leading to DNA DSBs induced by diverse treatments and conditions not previously realized to target Top1. Further studies investigated 8OxoG^[13]^, benzo[*a*]pyrene diol epoxide adducts^[19]^, and other types of damage^[20]^, and demonstrated these either trapped Top1 or inhibited Top1 cleavage, in a position-dependent manner. The paradoxical position-dependent enhancement/inhibition effects of damaged bases on Top1 activity were investigated using X-ray structural analysis. These studies revealed Top1 binds DNA in an inactive conformation and rearrangement of the active site is required for catalysis. It was found that 8-OxoG at the +1 position of the scissile strand stabilized the inactive, DNA-bound state^[21]^.

## POISONING OF TOP1 WITH NUCLEOSIDE ANALOGS

**Figure 1.**
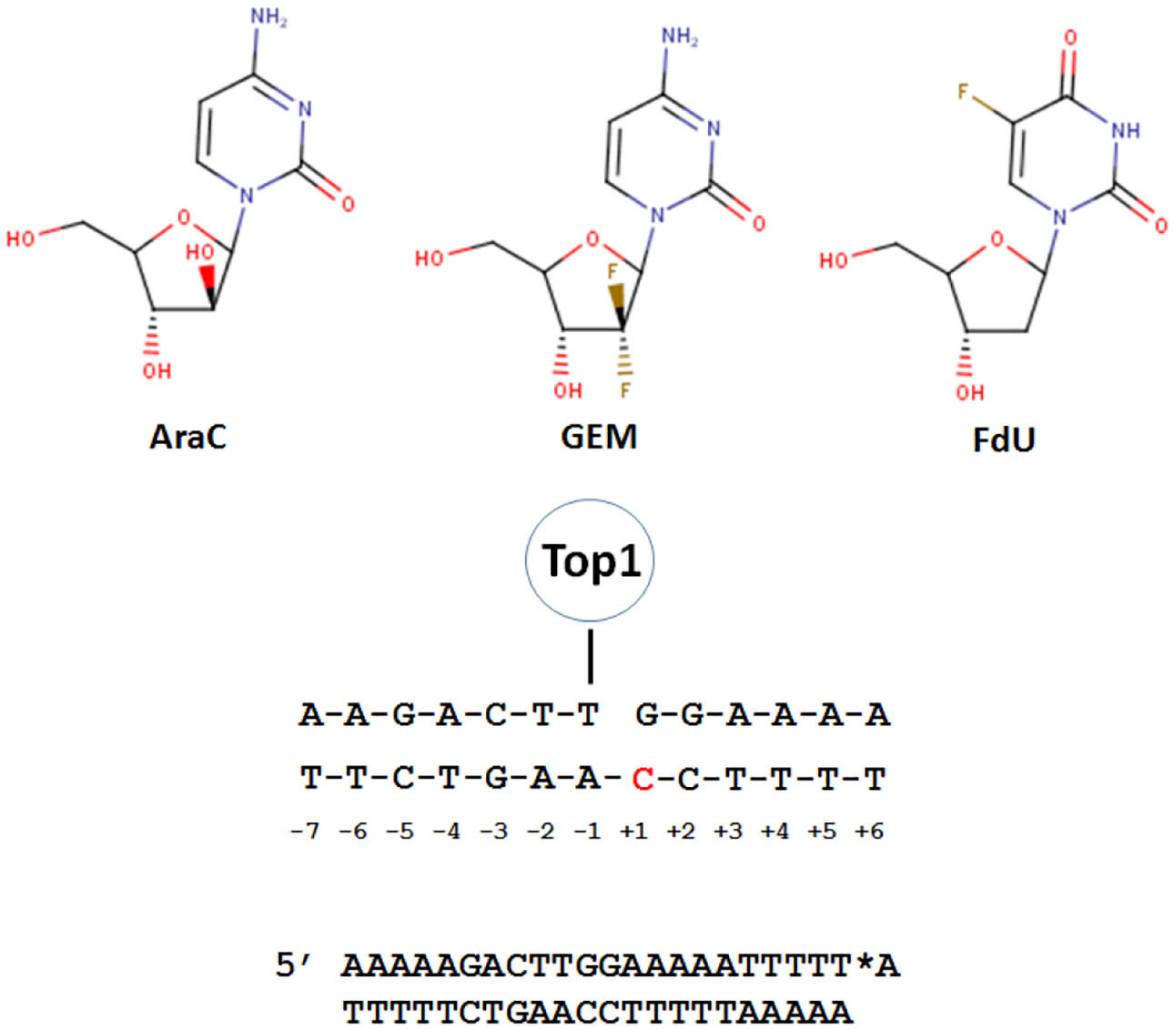
Substitution of nucleoside analogs into a model Top1 cleavage site results in Top1cc. (Top) The chemical structures for three nucleoside analogs that induce Top1cc in genomic DNA; (Middle) sequence of a model Top1cc used to investigate nucleoside analog substitution effects on Top1cc with the +1 site in the non-scissile strand highlighted; and (Bottom) 23mer model Top1cc substrate used to study cleavage/re-ligation effects of nucleoside analogs. *A corresponds to the [^32^P]-cordycepin label. Modified from References^[27,39,46]^. Top1: topoisomerase 1; AraC: cytarabine; GEM: gemcitabine; FdU: 5-fluoro-2’-deoxyuridine; Top1cc: Top1 cleavage complex

Nucleoside analogs such as cytarabine (AraC) are among the most active anti-cancer drugs^[22]^, and are used to treat diverse malignancies including front-line treatment for acute myeloid leukemia. AraC is phosphorylated [by deoxycytidine kinase (dCK)], and AraCTP (AraC 5’-*O*-triphosphate) is incorporated into nascent DNA during replication, which alters DNA conformation^[23]^ and stability^[24]^ and inhibits strand elongation. AraC thus serves as a chain-terminating nucleoside analog^[25]^. Studies with the same model system derived from Tetrahymena hexadecameric rDNA sequence described by Westergaard and co-workers^[26]^ were used to study the effects of DNA damage on Top1 activity [Figure 1]. These studies revealed that AraC at the +1 position relative to the Top1 cleavage site enhanced Top1cc formation 4-6- fold by inhibiting the re-ligation step of Top1 catalysis^[27]^. AraC substitution at the scissile and non-scissile strands had similar effects on Top1cc enhancement [Table 1]. The biological relevance of Ara-Cinduced Top1cc was demonstrated using CPT-resistant murine leukemia cells which were 7–10-fold more resistant to AraC than parental cells, but equally sensitive to alkylating agents, Top2 inhibitors, and tubulin poisons. The levels of Top1cc formed in AraC-treated cells were lower than those due to CPT treatment, likely because AraC’s chain termination property self-limits its incorporation into DNA. This may explain why recent immunodetection studies did not detect Top1cc in AraC-treated cells^[28]^. AraC incorporation into DNA also poisons Top2^[29]^. The DNA damage response activated by AraC is similar to Top1 and Top2 poisons and involves Rad9 binding and S-phase checkpoint activation^[30]^. DNA synthesis inhibitors including AraC display similar dependence as Top1 and Top2 poisons to multiple DNA repair genes including *SLFN11* and *SLX4*^[31]^. SLFN11 was discovered in a bioinformatics analysis^[32]^, and is crucial for mediating the cytotoxicity of Top1 and Top2 poisons, including nucleoside analogs such as AraC that cause Top1-mediated DNA damage^[33]^. Interestingly, AraC blocking elongation of 3’-termini is removed by tyrosyl DNA phosphodiesterase 1 (Tdp1), an enzyme that also cleaves the tyrosyl-DNA phosphodiester bond during Top1cc repair. Tdp1 also repairs DNA breaks induced by Sapacitabine, a nucleoside analog with structural similarities to AraC^[34]^.

Gemcitabine (GEM^[35]^ or dFdC) is a deoxycytidine (dC) analog that is used for front-line treatment of pancreatic cancer and other malignancies. Structurally, gemcitabine differs from dC by inclusion of a geminal difluoro at C2’. GEM is readily phosphorylated by dCK and the diphosphate (dFdCDP) inhibits ribonucleotide reductase, depleting cellular dCTP and potentiating dFdCTP incorporation into DNA. Upon incorporation of GEM into DNA, polymerase pausing occurs, but, unlike AraC, GEM is not primarily chain terminating and most GEM is present at internucleotide positions in genomic DNA^[36]^. GEM cytotoxicity strongly correlates with DNA incorporation. Gmeiner and co-workers showed that GEM inclusion in a model Okazaki fragment altered the electrostatic surface, which may affect protein binding^[37,38]^. Inclusion of GEM into two model Top1 substrates revealed position-specific effects on Top1cc formation^[39]^. GEM substitution at the +1 site increased Top1cc formation 5–7-fold while GEM at the −5 position of the non-scissile strand induced a new Top1 cleavage site adjacent to GEM substitution. GEM effects were site-specific and GEM at −3 or +2 of the scissile strand had no effect on Top1cc formation. Similar to AraC, GEM inhibited the re-ligation step of Top1 catalysis and Top1-deficient P388 CPT-resistant murine leukemia cells were cross-resistant to GEM, consistent with the *in vivo* significance of Top1cc for anti-tumor activity.

## DUAL TARGETING OF THYMIDYLATE SYNTHASE/TOP1 WITH F10

Fluoropyrimidine drugs (FPs) are used to treat > 2 million cancer patients worldwide each year^[40]^. Although the deoxynucleotide metabolites FdUMP and FdUTP are primarily responsible for anti-cancer activity, FPs are administered either as the nucleobase (5-FU) or as 5-FU pro-drugs (e.g., capecitabine), because the deoxynucleoside FdU is very rapidly converted to 5-FU in the liver^[41]^. A principal target for FP chemotherapy is thymidylate synthase (TS)^[42]^, which is required for *de novo* thymidine (Thy) biosynthesis to support rapid proliferation of malignant cells. 5-FU is inefficiently converted to the TS inhibitory metabolite FdUMP with ~85% of the dose administered to humans degraded or excreted intact^[43]^. Among anabolic metabolites, ribonucleotides that contribute primarily to systemic toxicities are produced at higher levels than deoxynucleotides^[44]^. The Gmeiner lab developed FP polymers (e.g., F10) to efficiently generate FdUMP^[45]^. F10 displayed improved cytotoxicity relative to 5-FU in the NCI60 cell line panel^[46,47]^, is preferentially taken up by malignant cells^[48]^, and thus does not require extracellular degradation to monomers for biological activity.

Analysis of F10’s mechanism based on the response profile across the NCI60 cell line screen using the COMPARE^[49]^ algorithm revealed similarities to Top1 poisons^[46,47]^. In contrast, 5-FU was distantly related to both F10 and Top1 poisons. In collaboration with Pommier, we showed F10 induced Top1cc in malignant cells and that CPT-resistant cells were cross-resistant to F10 as well as to FdUMP, FdU, and raltitrexed (an anti-folate TS inhibitor)^[46]^. FdU mismatched base pairs in the non-scissile strand at positions +1 or +2 relative to a model Top1 cleavage site trapped Top1cc, and inhibited the re-ligation step of Top1 catalysis. These studies used the same model duplex used to study Top1cc by AraC and GEM [Figure 1]. We went on to show that FdU-dG mismatched base pair destabilized duplex DNA, which may explain why FdU substitution in the non-scissile strand perturbed Top1-mediated re-ligation^[50,51]^. The cytotoxic effects of F10 require TS inhibition and are reversible with exogenous Thy; however, Thy reversibility is limited to < 16 h of treatment^[52]^, a time that corresponds to Top1cc formation, after which F10’s cytotoxic effects are no longer reversible with Thy. Thus, Top1cc formation appears to be an irreversible step in F10’s mechanism leading to cell death.

**Figure 2.**
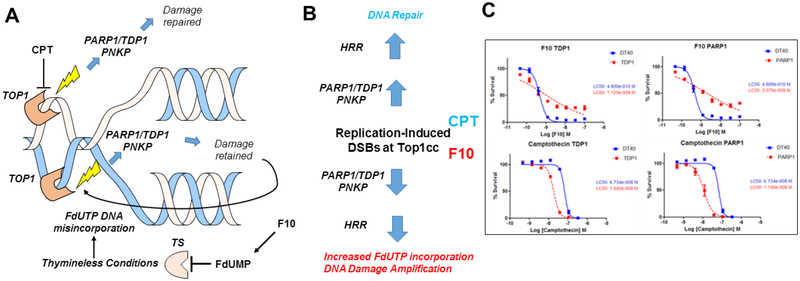
F10 and CPTs both form Top1cc but have different outcomes in response to PARP1/TDP1-mediated repair. A: CPTs form ternary Top1cc that are repaired thru proteasomal degradation to a peptide stub, TDP1-mediated cleavage of the peptide: DNA bond, and PNKP-mediated modulation of 5′-end-phosphorylation status followed by ligation to restore the duplex. In contrast, this same enzymatic process retains FdU in DNA and is susceptible to further Top1cc formation at the same site; B: Activation of homologous HRR due to incomplete TDP1-mediated repair stimulates DNA resection and re-synthesis, which under thymineless conditions increases FdUTP incorporation into DNA, stimulating further Top1-mediated DNA damage; C: In DT40 cells, F10 and CPTs display opposite dependence on TDP1 and PARP1 expression^[53]^. CPT: camptothecin; Top1: topoisomerase 1; HRR: recombination repair; TDP1: tyrosyl DNA phosphodiesterase 1; TS: thymidylate synthase; Top1cc: Top1 cleavage complex

Top1cc induced by F10 differ fundamentally from CPT analogs because repair occurs under thymineless conditions, which renders repair ineffective since FdU or dU are re-incorporated at the lesion site [Figure 2A and B]. Using DT40 knockout (ko) cells deficient in Tdp1, PARP1, and other DNA repair enzymes, we showed that F10 and CPT displayed an opposite dependence on the expression of Tdp1^[53]^ [Figure 2C], an important Top1cc repair enzyme. While Tdp1-ko cells were hypersensitive to CPT, they were relatively resistant to higher concentrations of F10, which is consistent with Tdp1 contributing to F10 cytotoxicity. Results for PARP1-ko cells mirrored Tdp1, which is consistent with PARP1/Tdp1 epistasis for Top1cc repair^[54]^. A possible explanation for these differences is that Tdp1-mediated repair actually amplifies F10-induced Top1-mediated DNA damage by repeatedly regenerating the lesion [Figure 2A and B]. This dual targeting of TS/Top1 by F10 results in potent anti-tumor activity, which we have demonstrated occurs in multiple pre-clinical models of AML^[55]^, acute lymphocytic leukemia^[48]^, prostate cancer^[56]^, glioblastoma^[57]^, colorectal cancer, and pancreatic ductal adenocarcinoma.

## TOP1 POISONS AND REPLICATION STRESS

Nucleoside analogs and CPT derivatives both generate Top1cc and, while nucleoside analogs affect alternative targets (e.g., polymerase pausing and chain termination), collectively these agents are part of a larger class of drugs that exert anti-cancer activity primarily through replication stress^[31]^. Studies in recent years have begun to elucidate the molecular factors that generally affect cellular response to this class of agents. The goal of these studies is to identify which patients are likely to respond to therapy with these agents, and to identify new molecular targets that complement their Top1-directed activities. PARP and Tdp1 are both important for Top1cc repair and inhibitors of these enzymes are being evaluated in combination with Top1 poisons, with PARP inhibitors showing promising activity in this context in recent clinical studies^[58]^. Recent studies have identified SLFN11 as the major determinant in response to drugs that induce replication stress^[33]^. Malignant cells that downregulate SLFN11 through epigenetic silencing are relatively less sensitive to Top1 poisons and other agents that induce replication stress. Either agents that reverse epigenetic silencing or those that inhibit ATR, a kinase that functions in parallel with SLFN11 in repressing replication in cells treated with Top1 poisons, can sensitize cells with downregulated SLFN11 expression to the cytotoxic activities of Top1 poisons^[59]^. Efficacy of combination therapy regimens will depend on both malignancy-specific factors such as SLFN11 expression and drug-specific factors. In particular, the opposite dependence of F10 and CPT with regards to PARP/Tdp1-mediated repair indicates that different approaches may be required to enhance the activities of fluoropyrimidine polymers relative to CPT derivatives, or alternative nucleoside analogs. Top1-directed activities of nucleoside analogs contribute to their clinical activities. Comprehensive, mechanism-based combination therapy can enhance these activities, and further improve outcomes.

## CONCLUSION

Top1 poisons continue to be among the world’s most important anti-cancer drugs. In addition to being the sole target for CPT derivatives, Top1 is an important target for agents that induce DNA damage including ionizing radiation, reactive oxygen species, or formation of covalent adducts. Top1 is also target for nucleoside analogs such as AraC and GEM. Dual targeting of TS/Top1 by DNA-directed fluoropyrimidines such as F10 shows promising activity in pre-clinical cancer models. TS/Top1 dual targeting is distinct from Top1 poisoning by CPT derivatives because Top1cc repair occurs under thymineless conditions and shows different dependence on Top1cc repair pathways. A challenge in coming years will be how to use the Top1 poisoning activity of nucleoside analogs for improved care in the context of personalized therapy taking into account patient-specific expression of DNA repair pathways.

## Figures and Tables

**Table 1.** Summary of nucleoside analogs and other DNA damage effects on Top1cc

Substitution	Effects on Top1cc	Ref.
Abasic site	Position-dependent, stable Top1cc in absence of CPT or suppression of Top1 cleavage	[16,17]
8-Oxo-dG	Position-dependent effects +1 scissile strand stabilized inactive Top1	[13,21]
BaP	Position-dependent effects	[19]
AraC	AraC in either scissile or non-scissile strand enhanced Top1cc; CPT-resistant cells are cross-resistant to AraC	[27]
GEM	Position-dependent effects including inducing new Top1cc sites; CPT-resistant cells are cross-resistant to GEM	[39]
FdU	FdU substitution +1 or +2 in non-scissile strand inhibited re-ligation step of Top1 catalysis; Top1cc important for F10 biological activity; opposite effect of Tdp1 knockout relative to CPT	[46,48,52,53,55,57]

CPT: camptothecin; Top1: topoisomerase 1; AraC: cytarabine; GEM: gemcitabine; FdU: 5-fluoro-2′-deoxyuridine; Tdp1: tyrosyl DNA phosphodiesterase 1; Top1cc: Top1 cleavage complex
